# Pharmacological Block of Genicular Nerves in the Treatment of Knee Osteoarthritis

**DOI:** 10.1055/s-0044-1792117

**Published:** 2024-12-21

**Authors:** Bruno Paulo Marques da Fonseca, Gilberto Yoshinobu Nakama, Guilherme Loterio Marques, Guilherme Ferrari de Araujo, Fernanda Martinho Soares, Alan Motta do Canto

**Affiliations:** 1 Departamento de Ortopedia, Hospital e Maternidade Metropolitano, São Paulo, SP, Brasil; 2 Departamento de Ortopedia e Cirurgia do Joelho, Instituto Prevent Senior, São Paulo, SP, Brasil

**Keywords:** nerve block, osteoarthritis, knee, pain

## Abstract

**Objective** To evaluate the clinical and functional outcomes of the pharmacological block of the genicular nerves as a modality in the therapeutic arsenal for knee osteoarthritis, since it is simple, safe, and minimally invasive.

**Methods** The pharmacological block of the genicular nerves was performed in 20 patients with grades 3 and 4 knee osteoarthritis per the Kellgren-Lawrence classification. We assessed their clinical and functional outcomes one, three, and six months after the procedure.

**Results** Of the 20 patients undergoing the procedure, 16 (80%) presented an excellent response in the first month of outpatient follow-up, since their pain level went from 8/9 to 2/3; 2 subjects presented a partial response, and 2 others did not respond to the treatment.

**Conclusion** The pharmacological block of the genicular nerves is efficient in reducing pain and improving the performance of daily activities by the patients, especially up to three months after the procedure.

## Introduction

Knee osteoarthritis (KOA) is a multifactorial degenerative disorder mainly affecting elderly subjects; it results in functional loss, pain, and joint stiffness. The diagnosis relies on anamnesis and physical examination, and its confirmation requires a weight-bearing knee radiography. The treatment for KOA requires an individualized approach for each patient, considering age, associated comorbidities, osteoarthritis degree (per the radiographic classification), and physical activity level. These data help to determine whether to proceed with the surgical or non-surgical treatments. 1 2

Non-surgical alternatives include the non-pharmacological treatment, which includes weight loss, motor physical therapy, muscle strengthening, orthoses, and braces,, and the pharmacological treatment, which includes nonsteroidal anti-inflammatory drugs (NSAIDs), topical agents (capsaicin), chondroprotective agents (glucosamine sulfate and diacerein), unsaponifiable extracts of soybean and avocado, duloxetine, and intra-articular injections of corticosteroids and hyaluronic acid. 3 In addition, prolotherapy and platelet-rich plasma (PRP) have been described and have gained ground with the advancements in regenerative medicine. 4

Genicular nerve pharmacological block (GNPB) has an efficacy similar to that of radiofrequency (RF) ablation in treating KOA-related chronic pain. In addition, as an advantage, GNPB is relatively simple and does not require a device emitting electromagnetic waves. 5 6

Despite the need for further studies, this technique has increasingly been present in the therapeutic arsenal of knee orthopedists because it is safe, simple, and minimally invasive. In addition, it can be performed in cases of KOA with no indication for prosthesis; it contributes to reduce chronic medication use, and it helps in the adjuvant therapy of motor physical therapy.

Therefore, the present study aimed to assess the efficacy of GNPB in controlling chronic knee pain and as a modality in the treatment of KOA.

## Materials and Methods

The institutional Ethics Committee approved the study under number CAAE: 54161921.7.0000.8054

The present study is a case series of 20 patients diagnosed with KOA and treated at the knee outpatient clinic of a hospital in the state of São Paulo who underwent GNPB from March to October 2021. The inclusion criteria were patients with moderate-to-severe KOA-related knee pain (numerical intensity of 6 or more), persistent pain for 6 months or more (chronic condition), moderate-to-advanced KOA (grade ≥ 3 in the Kellgren-Lawrence [KL] classification), and conservative treatment failure using analgesics, NSAIDs, and physical therapy. We documented the degree of knee deformity, the comorbidities, and the body mass index (BMI) of the patients. The exclusion criteria were patients with knee pain due to other causes (meniscopathy, trauma, spinal diseases, rheumatoid arthritis), previous surgeries in the region, intra-articular injection in the last 3 months, coagulopathies, those using anticoagulant and antiplatelet agents, and patients with infection or severe psychiatric illnesses.

**Fig. 1:**
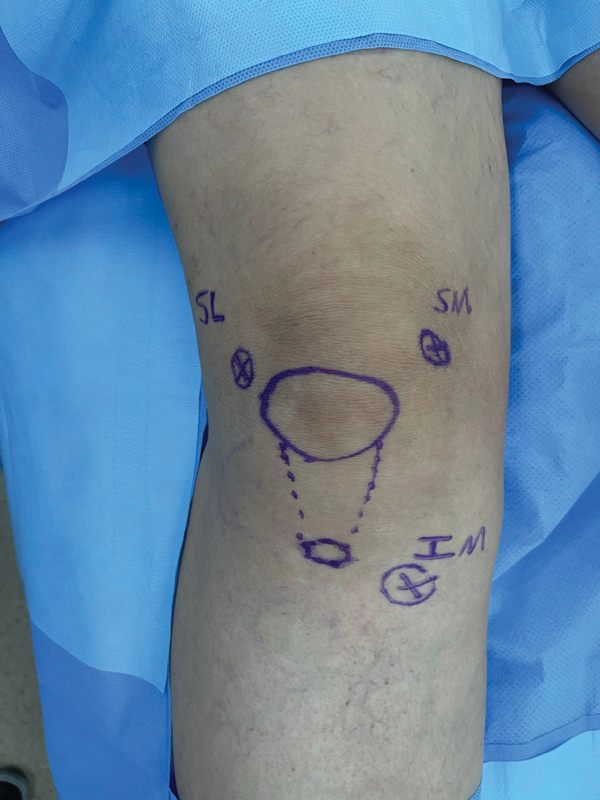
Target points marked on the skin of the right knee. **Abbreviations:** IM, inferomedial branch; SL. superolateral branch; SM, superomedial branch.

All GNPBs were performed in the surgical room under local anesthesia, sedation, and radioscopy to locate the anatomical points of the genicular nerves. After sedation, we placed the patient in horizontal dorsal decubitus and degermed the lower limb or limbs. After asepsis and antisepsis with alcoholic chlorhexidine and the placement of sterile fields, we obtained an anteroposterior (AP) image of the knee with the patella centered at the zenith with the aid of radioscopy. Next, we proceeded to local cutaneous and subcutaneous anesthesia with 2% lidocaine without vasoconstrictor at the 3 infiltration points. The first point was the site of the superolateral (SL) genicular nerve branch; the second was its superomedial (SM) branch. These two points are the edge of the cortex of the distal femur at the metaphysis-epiphysis transition, where the curvature of the femoral condyles begins. The third point was in the medial proximal tibia, at the inferomedial (IM) branch of the saphenous nerve ( Fig. 1 ). We avoided the proximal lateral region of the tibia due to the proximity of the common fibular nerve and its motor branches.

**Fig. 2:**
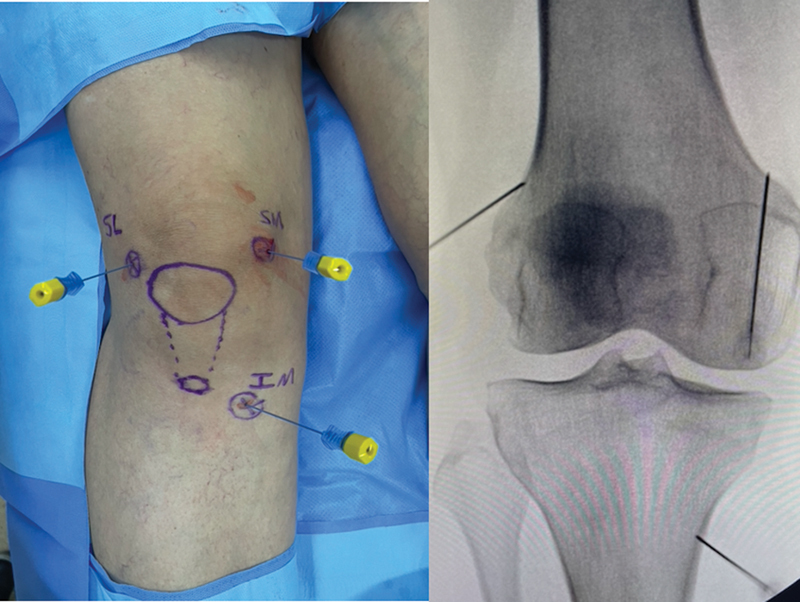
Correlation of cannula positioning in the anterior and anteroposterior (AP) views.

After local anesthesia and marking with a dermographic pen, we prepared a solution of 2 mL of methylprednisolone acetate (40 mg) plus 8 mL of ropivacaine without vasoconstrictor in a 10 mL syringe. We used 22 G × 100 mm blocking cannulas to infiltrate the solution into the target points. We positioned each cannula close to the cortical bone and perpendicular to the skin ( Fig. 2 ).

**Fig. 3:**
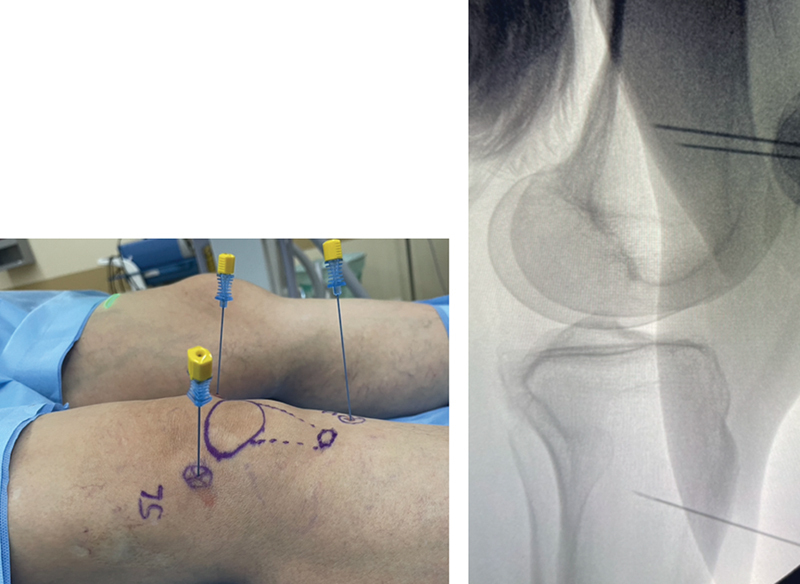
Correlation of cannula positioning in the lateral and profile views.

Next, through radioscopy on the lateral view, we checked the positioning of the cannula, whose tip should be in the center or slightly posterior to the middle of the femoral diaphysis. In the tibia, this point often corresponds to the level of the anterior tuberosity observed on the lateral view ( Fig. 3 ). After confirming the positioning with radioscopy, we injected 2 to 3 mL of the solution into each target point.

**Fig. 4:**
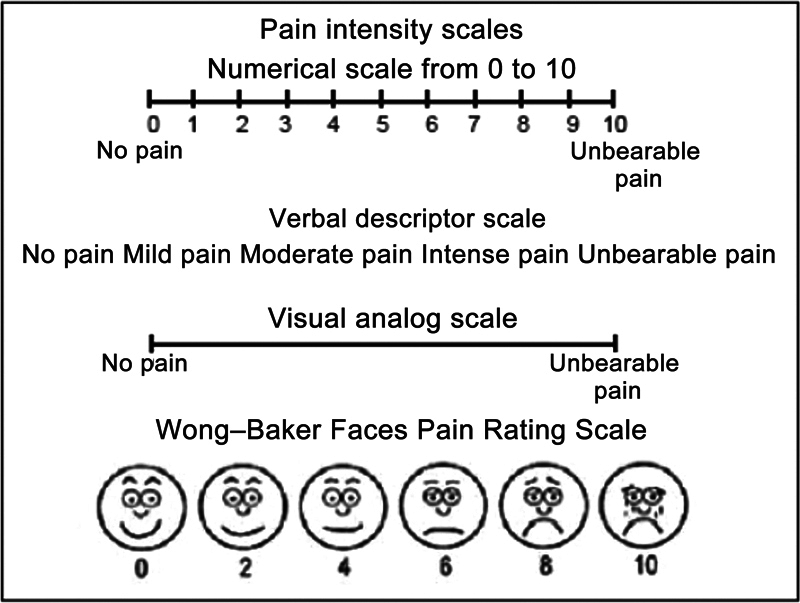
Visual Numeric Pain Scale.

After the procedure, the patients waited approximately 2 to 3 hours before discharge. We instructed all subjects to move their knee fully and walk with full weight-bearing. We scheduled outpatient follow-up visits at 1, 3, and 6 months after the procedure. We documented pre and postprocedure clinical parameters of all cases according to the Visual Numeric Scale (VNS; Fig. 4 ). There were no postprocedural complications.

We instructed all patients not to undergo any other treatment (except for physical therapy and knee strengthening) after the procedure to avoid interfering with the results.

## Results

Regarding the demographic data, the age of the patients ranged from 49 to 92 (mean: 63.5) years, and most were elderly individuals who presented with chronic knee pain. In addition, most patients were female (75%), and all had moderate-to-advanced KOA (KL ≥ 3; Table 1 ).

**Table 1:** Characteristics of the study sample

Case	Age	Gender	Comorbidities	Previous treatment	KL	BMI	Deformity
1	64	Female	Healthy	PT and NSAIDs	3	22.0	Varus
2	58	Female	SAH	PT and NSAIDs	3	24.9	Varus
3	77	Male	SAH and underweight	PT and NSAIDs	4	18.3	Valgus
4	49	Female	Obesity, SAH, and DM	PT and NSAIDs	5	41.0	Varus + flexion
5	55	Female	SAH and overweight	PT and NSAIDs	3	25.6	Varus
6	63	Male	Healthy	PT and NSAIDs	3	20.5	Varus
7	62	Female	DM and obesity	PT and NSAIDs	3	36.9	Varus
8	56	Female	SAH and obesity	PT and analgesic agents	4	38.4	Valgus + recurvatum
9	65	Female	Obesity	PT and analgesic agents	3	31.0	Varus + flexion
10	52	Female	Overweight	PT and analgesic agents	3	25.1	Varus
11	71	Female	SAH, DM, and overweight	PT andanalgesic agents	3	26.5	Valgus
12	92	Female	Overweight, SAH, and DM	PT and analgesic agents	5	29.9	Varus + flexion
13	47	Female	SAH and overweight	PT and analgesic agents	3	28.4	Varus
14	84	Female	SAH and obesity	PT and analgesic agents	4	32.9	Varus
15	88	Female	DM and underweight	PT and analgesic agents	4	17.1	Varus
16	80	Female	Overweight and SAH	PT and analgesic agents	4	29.4	Valgus
17	48	Male	Healthy	PT and analgesic agents	3	23.7	Neutral knee
18	75	Female	Overweight and DM	PT and analgesic agents	3	28.8	Valgus
19	80	Female	SAH and overweight	PT and analgesic agents	4	27.8	Varus
20	44	Female	SAH	PT and analgesic agents	3	24.6	Varus

**Abbreviations:** BMI, body mass index; DM, diabetes mellitus; KL, Kellgren-Lawrence classification for knee osteoarthritis; NSAIDs, non-steroidal anti-inflammatory drugs; PT, motor physical therapy; SAH, systemic arterial hypertension.

The BMI ranged from 17 to 41 (mean: 30.87) Kg/m ^2^ ; 2 patients were underweight (BMI < 18.5 Kg/m ^2^ ), 5 were eutrophic (BMI between 18.5 and 24.9 Kg/m ^2^ ), 8 were overweight (BMI between 25 and 29.9 Kg/m ^2^ ), 5 were obese (BMI ≥ 30 Kg/m ^2^ ), and 1 was morbidly obese (BMI > 40 Kg/m ^2^ ). As for the deformities, 11 patients presented varus, 3, varus and flexion, 4, valgus, 1, valgus and recurvatum, and 1 presented a neutral knee.

Of the 20 patients undergoing the procedure, 16 (80%) had an excellent response until the first month's outpatient follow-up, and their pain level went from 8/9 to 2/3; 2 had a moderate response, and 2 others did not respond to the treatment.

In the third month after the procedure, of the 16 (80%) patients with an excellent response, 8 (40%) maintained a minimum pain level, as they had adhered to the adjuvant treatment of motor physical therapy and weight loss. The other 8 (40%) subjects presented pain recurrence. The 2 patients with a moderate response maintained the same pain level. The 2 subjects who did not respond were referred for open surgery for total knee arthroplasty.

## Discussion

Knee osteoarthritis affects approximately 6% of adults over 30 years old, and its prevalence increases with age. This condition accounts for significant morbidity, leading to pain and functional disability in more than 3.6% of the global population. Approximately 14% of patients with KOA require assistance to perform their daily activities, while 11% require help with daily personal care. 7

The initial treatment for KOA is conservative, and it involves approaches ranging from guidance and non-pharmacological measures (weight loss and physical therapy) to medications and surgical procedures (blocks, infiltrations, and open surgeries, such as osteotomies and arthroplasties).

Genicular nerve block is frequently used in the conservative KOA treatment, especially when initial measures fail or pain persists. This procedure has several variations regarding technique, form, and nerve block agent.

The nerve block procedure can be temporary or permanent, and it may be chemical or physical. Moreover, the imaging method that will help locate the target points for the block may very: ultrasound or radioscopy. Both provide indirect parameters for genicular nerve location.

Ultrasound enables the location of the genicular artery as a parameter, and the nerve endings will be very close to it. Its advantage is the dynamic visualization of the cannula tip in real-time to determine the volume of the solution for injection. A potential disadvantage is that the device is operator-dependent and requires specific training.

In the present study, due to the availability and experience of the surgeons, the method of choice to perform all procedures was radioscopy. Although the genicular nerves and their branches present great anatomical variation, several authors, such as Fonkoue et al., 8 have increased the accuracy of the procedure by locating the target points previously described. Furthermore, Kim et al. 9 observed no significant clinical differences or complications when comparing ultrasound with radioscopy in genicular nerve block.

In a systematic review, Tan et al. 10 demonstrated the efficacy of the GNPB with anesthetics, steroids, and alcohol (phenol) using ultrasound. The authors observed pain reduction and knee functionality improvement in osteoarthritis cases, but reported that further studies were needed for a better interventional approach.

Steroid application can occur intra- or extra-articularly (close to the nerves) for KOA treatment. Among the several current examples, the best known and used consists of triamcinolone, methylprednisolone, betamethasone, and dexamethasone. It is worth emphasizing that all these options have variable deposition potential, time of action, and duration. Studies have compared the use of intra-articular steroids in OA treatment. Donovan et al. 11 compared intra-articular steroid injection with placebo and observed an improvement in symptoms in the experimental group for 3 months. In 1987, Balogh and Ruzsonyi 12 noticed greater efficacy and longer duration triamcinolone of compared to betamethasone.

Yavuz et al. 13 compared the efficacy of intra-articular injections of methylprednisolone acetate, betamethasone disodium phosphate, triamcinolone, and saline solution for KOA treatment over 12 weeks. The three steroids provided symptomatic and functional improvement, but methylprednisolone acetate was more effective in relieving pain up to the sixth week of evaluation. Triamcinolone and methylprednisolone are the most used and studied agents in the clinical practice. 14 15 Prednisolone and triamcinolone are less soluble than other steroids for intra-articular injection. The addition of the methyl and acetate groups to these steroids further reduces their solubility and prolongs their action. 16 Adding a fluorine atom to form triamcinolone increases the steroid potency, and adding hexacetonide prolongs its action. However, the potential for subcutaneous cellular tissue atrophy is higher in case of extravasation or extra-articular injection. Although they are the most frequently employed drugs, Pyne et al. 17 observed greater efficacy in the intra-articular application of triamcinolone in the third week and of methylprednisolone in the eighth week, which prevented them from obtaining an ideal comparative value.

In 2015, Lomonte 18 compared the efficacy of intra-articular infiltration of triamcinolone hexacetonide versus methylprednisolone acetate in KOA in a 24-week, double-blinded, and randomized study. The author concluded that intra-articular infiltrations of triamcinolone and methylprednisolone acetate are equally effective in controlling pain in patients with KOA in the fourth week after the procedure. They also noticed that the benefits would remain for up to 24 weeks regardless of the steroid administered.

In addition to steroids, other substances or methods may play a role in GNPB and pain control. Yildiz et al. 19 compared the efficacy of genicular neurolysis with phenol or radiofrequency ablation in patients with KOA, noting that both techniques improved pain scores in subjects undergoing the procedure, with no statistically significant differences.

Although the results are similar, phenol and radiofrequency have some differences. Phenol neurolysis is a simple, low-cost procedure performed under ultrasound guidance. It is often quick and painless due to the anesthetic action of phenol. In contrast, radiofrequency is more complex and expensive, and it requires specialized equipment and training. 19

A potential disadvantage of phenol is the high risk of nerve injury or neuritis because it can extravasate beyond the target area, affecting other structures. González Sotelo et al., 20 for example, observed phenol in the posterior region of the knee after applying 4 mL to the genicular nerve.

In the literature, the recommended dosage for phenol application ranges from 0.5 to 2 mL per genicular nerve. However, even considering these limits, Yildiz et al. 19 found a higher postapplication paresthesia rate than that of radiofrequency; they reported that paresthesia was self-limited, lasting up to 15 days, not requiring treatment.

In addition to the cost-related disadvantage, radiofrequency ablation procedures have been described 19 as more time-consuming and painful due to nerve ablation. It can cause complications such as skin burns or infections because it involves skin punctures and electrode insertions. Its great advantage over other methods is that it can last up to 1 year. 21

Although the present study showed positive GNPB outcomes, some patients did not show a positive response. A possible explanation is the infiltration only in the three well-defined main branches of the genicular nerves. Moreover, we did not perform an adductor canal block. More than seven nerves have been identified as innervating the knee, and some patients may benefit from an associated intra-articular block. 22

Another potential explanation is that radioscopy does not locate the main nerve branch, which could be a disadvantage compared to ultrasound. 23 Due to this fact and the absence of radiation emission, Yildiz et al. 19 recommend ultrasound as an alternative to fluoroscopy.

It is also critical to emphasize that the 2 patients who did not respond to treatment (cases 4 and 12) had a maximum KL osteoarthritis degree (5), as well as varus plus flexion deformity. In addition, one of the patients was extremely obese (BMI > 40 kgm ^2^ ) at the time. The sum of these variables may have contributed as a confounding factor in our study and the null therapeutic response to treatment, as shown in Tables 1 and 2 . Therefore, in patients with severe and combined deformities or a very high BMI, this procedure should be viewed with caution due to the possibility of later failure.

**Table 2:** Pre- and postprocedural scores on the Visual Numeric Scale (VNS)

Cases	Preoperative VNS	VNS 1 month after the procedure	VNS 3 months after the procedure	VNS 6 months after the procedure
1	8	2	3	4
2	7	2	4	7
3	8	2	3	8
4	8	8	8	8
5	9	3	4	9
6	8	3	3	4
7	8	4	5	8
8	7	2	4	7
9	8	2	2	8
10	9	2	3	9
11	9	3	5	9
12	9	9	9	9
13	8	2	4	8
14	9	2	5	9
15	9	2	7	9
16	9	1	3	4
17	7	2	2	3
18	8	1	3	8
19	8	2	4	4
20	8	2	2	8

We also observed that in the sixth month after GNPB, 16 (80%) patients presented the same preprocedural pain. As previously mentioned, the chemical block is reversible and depends on the painful stimulus and the inflammatory cascade occurring at the chondral level. In these cases, it is essential to emphasize that all patients must undergo pre- and postprocedure physical therapy to improve muscle quality during treatment and ensure longer-lasting analgesia.

Although the present study has certain limitations, some considerations are worth highlighting. The GNPB procedure with local anesthetic and steroids was successful for most patients, who showed pain improvement for a prolonged period (over 3 months). This occurs because GNPB acts both locally and systemically by influencing central pain modulating pathways.

In addition, GNPB may be important initially as a test block, with the expectation of immediate pain improvement (due to the prompt action of the local anesthetic) and long-term (3 months) improvement. In cases with a favorable response, after this expected period, the professional may consider the test positive and indicate a more prolonged block, such as phenol neurolysis or radiofrequency ablation, for example.

## Conclusion

The GNPB procedure is an effective method to treat KOA-related chronic pain that results in pain relief according to the VNS. It can be an adjuvant to improve patient quality of life and a therapeutic tool to enable more effective physical therapy and physical activity, ameliorating KOA symptoms. It should be viewed cautiously in patients with advanced and combined deformities such as varus and flexion or BMI higher than 40 Kg/m ^2^ .
