# Supplementary material for: Pharmacological Block of Genicular Nerves in the Treatment of Knee Osteoarthritis
Source: Rev Bras Ortop (Sao Paulo). 2024 Dec 21;59(6):e888–94. doi: 10.1055/s-0044-1792117 (PMC11663067; doi:10.1055/s-0044-1792117)
Supplement: Related texts

# Research article: Bloqueio farmacológico dos nervos geniculares no tratamento da osteoartrite de joelho

**Keywords:** bloqueio nervoso, dor, osteoartrite do joelho

## Abstract

**Objetivo** Avaliar os resultados clínicos e funcionais do bloqueio farmacológico dos nervos geniculares como modalidade no arsenal terapêutico da osteoartrite de joelho, uma vez que é simples, seguro e minimamente invasivo.

**Métodos** Foi realizado o bloqueio farmacológico dos nervos geniculares de 20 pacientes com osteoartrite de joelho de graus 3 e 4 segundo a classificação de Kellgren-Lawrence, e foram avaliadas as respostas clínica e funcional no primeiro, terceiro e sexto meses após o procedimento.

**Resultados** Dos 20 pacientes estudados e submetidos ao procedimento, 16 (80%) tiveram uma resposta excelente até o retorno ambulatorial do primeiro mês, isto é, foram de um nível de dor de 8/9 para 2/3; 2 deles tiveram uma resposta parcial e outros 2 não responderam ao tratamento.

**Conclusão** O bloqueio farmacológico dos nervos geniculares é eficiente na diminuição da dor e na melhora do desempenho dos pacientes nas atividades diárias, principalmente até o terceiro mês após o procedimento.

## Introdução

A osteoartrite do joelho (OAJ) é uma desordem degenerativa multifatorial que afeta principalmente os idosos, e leva a perda de função, dor e rigidez articular. O diagnóstico pode ser feito por meio de anamnese e exame físico, e é confirmado pela radiografia de joelho com carga. O tratamento da OAJ deve ser individualizado para cada paciente e levar em consideração, principalmente, a idade, as comorbidades associadas, o grau de artrose (com base na classificação radiográfica) e o nível de atividade física. Com esses dados, pode-se decidir pelos tratamentos cirúrgico ou não cirúrgico. 1 2

Quanto às alternativas de tratamento não cirúrgico, há o tratamento não medicamentoso, que inclui perda de peso, fisioterapia motora, fortalecimento muscular, órteses e joelheiras, e o tratamento medicamentoso, que inclui os anti-inflamatórios não esteroidais (AINES), os agentes tópicos, como a capsaicina, os condroprotetores, como o sulfato de glicosamina e a diacereína, os extratos insaponificáveis da soja e do abacate, além da duloxetina e das injeções intra-articulares de corticoide e ácido hialurônico. 3 Além disso, foram descritos a proloterapia e o plasma rico em plaquetas (PRP), que estão ganhando espaço com o avanço da medicina regenerativa. 4

O bloqueio farmacológico dos nervos geniculares (BFNG) tem uma eficácia semelhante à da ablação por radiofrequência (RF) para o tratamento de dor crônica na OAJ, e apresenta a vantagem de ser relativamente mais simples e não necessitar do aparelho emissor de ondas eletromagnéticas. 5 6

Apesar da necessidade de mais estudos, esta técnica tem se demonstrado cada vez mais presente no arsenal terapêutico do ortopedista de joelho, uma vez que é segura, simples e minimamente invasiva. Ademais, pode ser realizada nos casos de artrose do joelho sem indicação de prótese; contribui para diminuir o uso crônico de medicamentos e auxilia na terapia adjuvante de fisioterapia motora.

Portanto, o objetivo deste estudo foi avaliar a eficácia do BFNG no controle da dor crônica do joelho e como modalidade no tratamento de OAJ.

## Materiais e Métodos

Este trabalho foi aprovado pelo Comitê de Ética institucional sob o número CAAE: 54161921.7.0000.8054.

Trata-se de uma série de casos de 20 pacientes com diagnóstico de OAJ que foram atendidos no ambulatório de joelho em um hospital do estado de São Paulo e submetidos ao BFNG de março a outubro de 2021. Com relação aos critérios de inclusão, os pacientes apresentavam dor de moderada a severa no joelho devido à OAJ (intensidade numérica de 6 ou mais), dor persistente por 6 meses ou mais (crônica), OAJ moderada/avançada (de grau ≥ 3 na classificação de Kellgren-Lawrence [KL]) e falha no tratamento conservador com o uso de analgésicos, AINES e fisioterapia. O grau de deformidade do joelho, as comorbidades e o índice de massa corporal (IMC) também foram documentados. Foram excluídos do estudo pacientes com dor no joelho devido a outras causas (meniscopatia, trauma, doenças da coluna e artrite reumatoide), cirurgias prévias na região, injeção intra-articular nos últimos 3 meses, coagulopatias, uso de anticoagulantes e de antiagregantes plaquetários, infecção e doença psiquiátrica severa.

Todos os BFNGs foram realizados no centro cirúrgico com anestesia local, sedação e com o auxílio da radioscopia para localizar os pontos anatômicos dos nervos geniculares. Após sedação, o paciente foi colocado em decúbito dorsal horizontal, e o membro inferior ou ambos os membros inferiores foram degermados. Após assepsia e antissepsia com clorexidina alcoólica e a colocação de campos estéreis, uma imagem em incidência anteroposterior (AP) do joelho, com a patela centrada ao zênite, foi obtida com auxílio da radioscopia. Foi realizada anestesia local da pele e subcutâneo com lidocaína 2% sem vasoconstritor nos 3 pontos de infiltração. O primeiro ponto é o local do ramo superolateral (SL) do genicular; o segundo, o ramo superomedial (SM), ambos na borda da cortical do fêmur distal, na transição metáfise-epífise, onde começa a curvatura dos côndilos femorais; e o terceiro ponto foi feito na tíbia proximal medial, local do ramo inferomedial (IM) do nervo safeno ( Fig. 1 ). Evitou-se a região lateral proximal da tíbia devido à proximidade do nervo fibular comum e de seus ramos motores.

**Fig. 1:** Pontos-alvo marcados na pele do joelho direito. **Abreviaturas:** IM, ramo inferomedial; SL, ramo superolateral; SM, ramo superomedial.

Após a realização da anestesia local e da marcação com caneta dermográfica, preparou-se uma solução de 2 mL de acetato de metilprednisolona (40 mg) + 8 mL de ropivacaína sem vasoconstrictor numa seringa de 10 mL. Para infiltrar a solução nos pontos-alvo determinados, foram utilizadas cânulas de bloqueio de 22 G × 100 mm. Cada cânula foi posicionada rente à cortical óssea e perpendicular à pele ( Fig. 2 ).

**Fig. 2:** Correlação do posicionamento das cânulas na vista anterior e na incidência anteroposterior (AP).

Em seguida, verificou-se, por radioscopia na incidência em perfil do joelho, o posicionamento da cânula, cuja ponta deve estar centrada ou levemente posteriorizada em relação ao meio da diáfise femoral. Na tíbia, esse ponto geralmente corresponde ao nível da tuberosidade anterior observado no perfil ( Fig. 3 ). Confirmado o posicionamento, foram injetados de 2 a 3 mL da solução em cada ponto-alvo.

**Fig. 3:** Correlação do posicionamento das cânulas na vista lateral e na incidência em perfil.

Após o procedimento, os pacientes aguardaram cerca de 2 a 3 horas antes de receberem alta. Todos foram orientados a realizar movimentação completa do joelho e deambulação com carga total. Os retornos ambulatoriais foram agendados 1, 3 e 6 meses após o procedimento. Em todos os casos, foram documentados os parâmetros clínicos pré e pós-procedimento conforme a Escala Numérica Visual (ENV; Fig. 4 ), e não constatou-se nenhuma complicação após o procedimento.

**Fig. 4:** Escala Numérica Visual de dor.

Todos os pacientes foram orientados a não realizar nenhum outro tipo de tratamento (exceto fisioterapia e fortalecimento do joelho) durante o período pós-procedimento para não gerar interferência nos resultados.

## Resultados

Com relação aos dados demográficos, a idade dos pacientes variou de 49 a 92 (média: 63,5) anos , sendo que eles eram predominantemente idosos com dor crônica no joelho. A maioria era do sexo feminino (75%), e todos apresentavam OAJ de moderada a avançada (KL ≥ 3; Tabela 1 ).

**Tabela 1:** Características da amostra do estudo

Casos	Idade (anos)	Gênero	Comorbidades	Tratamento prévio	Grau na KL	IMC (Kg/m ^2^ )	Deformidade
1	64	F	Hígida	FST e AINES	3	22,0	Varo
2	58	F	HAS	FST e AINES	3	24,9	Varo
3	77	M	HAS e BP	FST e AINES	4	18,3	Valgo
4	49	F	OB, HAS e DM	FST e AINES	5	41,0	Varo + flexo
5	55	F	HAS e SB	FST e AINES	3	25,6	Varo
6	63	M	Hígido	FST e AINES	3	20,5	Varo
7	62	F	DM e OB	FST e AINES	3	36,9	Varo
8	56	F	HAS e OB	FST e ANALG	4	38,4	Valgo + recurvo
9	65	F	OB	FST e ANALG	3	31,0	Varo + flexo
10	52	F	SP	FST e ANALG	3	25,1	Varo
11	71	F	HAS, DM e SB	FST e ANALG	3	26,5	Valgo
12	92	F	SP, HAS e DM	FST e ANALG	5	29,9	Varo + flexo
13	47	F	HAS, SB	FST e ANALG	3	28,4	Varo
14	84	F	HAS e OB	FST e ANALG	4	32,9	Varo
15	88	F	DM e MG	FST e ANALG	4	17,1	Varo
16	80	F	SP e HAS	FST e ANALG	4	29,4	Valgo
17	48	M	Hígido	FST e ANALG	3	23,7	Joelho neutro
18	75	F	SP e DM	FST e ANALG	3	28,8	Valgo
19	80	F	HAS e SP	FST e ANALG	4	27,8	Varo
20	44	F	HAS	FST e ANALG	3	24,6	Varo

**Abreviaturas:** AINES, anti-inflamatórios não esteroidais; ANALG, analgésicos; BP, baixo peso; DM, diabetes melitus; F, feminino; FST, fisioterapia motora; HAS, hipertensão arterial sitêmica; IMC, índice de massa corporal; KL, classificação de Kellgren-Lawrence para osteoartrite de joelho; M, masculino; BP, baixo peso; OB, obesidade; SP, sobrepeso.

O IMC variou de 17 a 41(média: 30,87) Kg/m ^2^ : 2 pacientes apresentavam baixo peso (IMC < 18,5 Kg/m ^2^ ), 5 eram eutróficos (IMC de 18,5 a 24,9 Kg/m ^2^ ), 8 apresentavam sobrepeso (IMC de 25 a 29,9 Kg/m ^2^ ) e 5 apresentavam obesisade (IMC ≥ 30 Kg/m ^2^ ), sendo que 1 deles apresentava obesidade mórbida (IMC > 40 Kg/m ^2^ ). Com relação às deformidades, 11 pacientes apresentavam deformidade em varo, 3, em varo e flexo, 4, em valgo, 1, em valgo e recurvato, e 1, joelho neutro.

Dos 20 pacientes estudados e submetidos ao procedimento, 16 (80%) tiveram uma resposta excelente até o retorno ambulatorial do primeiro mês, isto é, foram de um nível de dor de 8/9 para 2/3; 2 deles tiveram uma resposta moderada, e outros 2 não responderam ao tratamento.

No terceiro mês após o procedimento, dos 16 (80%) pacientes que tiveram resposta excelente, metade (8; 40%) manteve o nível de dor mínima, pois conseguiu aderir ao tratamento coadjuvante de fisioterapia motora e perda de peso. A outra metade (8; 40%) apresentou retorno da dor. Os 2 pacientes que tiveram resposta moderada mantiveram o mesmo nível de dor. E os outros 2 pacientes, que não responderam ao tratamento, foram encaminhados para cirurgia aberta de artroplastia total do joelho.

## Discussão

A OAJ ocorre em aproximadamente 6% dos adultos acima de 30 anos, e sua prevalência aumenta com a idade. Essa patologia é responsável por significativa morbidade, que ocasiona dor e incapacidade funcional em mais de 3,6% da população mundial. Aproximadamente 14% dos pacientes com OAJ necessitam de auxílio para a realização de suas atividades diárias, ao passo que 11% necessitam de ajuda para cuidados pessoais do dia a dia. 7

O tratamento inicial da OAJ é conservador, envolve de orientações e medidas não medicamentosas (perda de peso e fisioterapia) a medicamentos e procedimentos cirúrgicos (bloqueios, infiltrações e cirurgias abertas, tais como osteotomias e artroplastias).

O bloqueio dos nervos geniculares é uma modalidade que tem sido frequentemente utilizada no tratamento conservador da OAJ, principalmente quando ocorre falha ou persistência da dor com as medidas iniciais. Com relação a esse procedimento, existem diversas variações a respeito da técnica, da forma e da substância usadas para o bloqueio dos nervos.

O procedimento de bloqueio dos nervos pode ser realizado de forma temporária ou definitiva, e o método utilizado pode ser químico ou físico. Além disso, pode-se variar o método de imagem que auxiliará na localização dos pontos-alvo de bloqueio: a ultrassonografia ou a radioscopia. Ambos fornecem parâmetros indiretos para a localização dos nervos geniculares.

Na ultrassonografia, localiza-se a artéria genicular como parâmetro, e as terminações nervosas estarão muito próximas. Sua vantagem é a de que a ponta da cânula está sendo visualizada dinamicamente em tempo real, e pode-se perceber o volume injetado da solução. Uma possível desvantagem é que o aparelho é dependente do operador e requer treinamento específico.

No presente estudo, devido à disponibilidade e à experiência dos cirurgiões, o método escolhido para todos os procedimentos foi a radioscopia. Apesar de os nervos geniculares e de seus ramos apresentarem uma grande variação anatômica, vários trabalhos, como o de Fonkoue et al., 8 contribuíram para aumentar a precisão do procedimento por meio da localização dos pontos-alvo descritos anteriormente. Além disso, Kim et al. 9 observaram que não há diferenças clínicas significativas e complicações na comparação do ultrassom com a radioscopia no bloqueio dos nervos geniculares.

Em uma revisão sistemática, Tan et al. 10 evidenciaram a eficácia do BFNG utilizando anestésicos, corticosteroides e álcool (fenol) com o auxílio da ultrassonografia. Os autores 10 observaram redução da dor e melhora da funcionalidade do joelho nos casos de osteoartrite, mas relataram que mais estudos são necessários para que se desenvolva uma abordagem intervencionista melhor.

Com relação aos corticosteroides, eles podem ser aplicados de forma intra ou extra-articular (próximo aos nervos) no tratamento da OAJ. Atualmente, existem vários exemplos, sendo os mais conhecidos e presentes no meio a triancinolona, a metilprednisolona, a betametasona e a dexametasona. É importante ressaltar que todos têm potencial de depósito, tempo de ação e duração variáveis. Estudos compararam o uso de corticoide intra-articular no tratamento da OA. Donovan et al., 11 que compararam o uso corticoide intra-articular e placebo, observaram uma melhora dos sintomas no grupo experimental por 3 meses. Em 1987, Balogh e Ruzsonyi 12 observaram maior eficácia e maior duração da triancinolona quando comparada à betametasona.

Yavuz et al. 13 compararam a eficácia das infiltrações intra-articulares de acetato de metilprednisolona, fosfato dissódico de betametasona, triancinolona e soro fisiológico na OAJ ao longo de 12 semanas. Os três corticosteroides promoveram melhora sintomática e funcional, mas o acetato de metilprednisolona foi mais eficaz no alívio da dor em relação aos outros agentes até a sexta semana de avaliação. Atualmente, a triancinolona e a metilprednisolona são os agentes mais comumente utilizados na prática clínica, e os mais frequentemente estudados. 14 15 A prednisolona e a triancinolona são menos solúveis em relação aos outros corticosteroides utilizados para a infiltração intra-articular. O acréscimo dos grupos metil e acetato aos respectivos corticoesteroides reduz ainda mais a sua solubilidade e prolonga sua ação. 16 Já o acréscimo de um átomo de flúor para formar a triancinolona aumenta a potência do corticosteroide, ao passo que o acréscimo da hexacetonida prolonga a sua ação. Entretanto, existe maior possibilidade de atrofia do tecido celular subcutâneo no caso de extravasamento ou de injeção extra-articular. Apesar de serem os mais frequentemente utilizados, Pyne et al. 17 observaram maior eficácia da aplicação intra-articular da triancinolona na terceira semana, ao passo que a metilprednisolona, na oitava semana, o que impediu a obtenção de um valor comparativo ideal.

Em 2015, Lomonte 18 comparou a eficácia da infiltração intra-articular de triancinolona hexacetonida e de acetato de metilprednisolona na OAJ em estudo duplo-cego e randomizado de 24 semanas. O autor concluiu que infiltrações intra-articulares de triancinolona e de acetato de metilprednisolona são igualmente eficazes no controle da dor em pacientes com OAJ na quarta semana após o procedimento, e, independentemente do corticosteroide usado, os benefícios podem ser mantidos por até 24 semanas.

Além dos corticosteroides, outras substâncias ou métodos podem ser utilizados para bloquear os nervos geniculares e controlar a dor. Yildiz et al. 19 compararam a eficácia da neurólise dos nervos geniculares com fenol e a ablação por radiofrequência em pacientes com OAJ, e observaram que ambas as técnicas foram capazes de melhorar as pontuações de dor nos indivíduos submetidos ao procedimento, sem diferenças estatisticamente significativas. 19

Embora haja similaridade nos resultados, existem algumas diferenças no uso do fenol e de radiofrequência. A neurólise por fenol é considerada um procedimento simples, que pode ser feito guiado por ultrassom e apresenta baixo custo. O procedimento geralmente é rápido e indolor devido à ação anestésica do fenol. Já o procedimento de radiofrequência é mais complexo e caro, e requer equipamento e treinamento mais especializados. 19

Uma possível desvantagem do fenol é o seu alto risco de lesão nervosa ou neurite devido à capacidade de extravasar além da área-alvo, afetando, assim, outras estruturas. González Sotelo et al., 20 por exemplo, observaram fenol na região posterior do joelho após aplicarem 4 mL no nervo genicular. 20

Na literatura, a dosagem recomendada de aplicação do fenol é de 0,5 a 2 mL por nervo genicular. Entretanto, mesmo respeitando esses limites, Yildiz et al. 19 encontraram maior taxa de parestesia após a aplicação em comparação com a radiofrequência; eles relataram que a parestesia é autolimitada e dura no máximo 15 dias, e não requer tratamento.

Além da desvantagem do custo, o procedimento de ablação por radiofrequência foi descrito 19 como mais demorado e doloroso devido à ablação nervosa. Além disso, pode causar complicações como queimaduras na pele ou infecções, pois envolve punções cutâneas e inserções de eletrodos. Sua grande vantagem em relação aos demais é que ele pode durar até 1 ano. 21

Embora o presente estudo tenha apresentado resultados positivos com o BNFG, alguns pacientes não apresentaram resposta positiva. Uma possível explicação é a de que, no procedimento realizou-se somente a infiltração dos três ramos principais e bem definidos dos nervos geniculares. Além disso, o bloqueio do canal dos adutores não foi realizado. Atualmente, sabe-se que mais de sete nervos foram identificados como responsáveis pela inervação do joelho e que alguns pacientes podem se beneficiar do bloqueio intra-articular associado. 22

Outra possível explicação é a de que, com o uso da radioscopia durante o procedimento, não se localiza o ramo nervoso principal, o que pode ser considerada uma desvantagem potencial em relação à ultrassonografia. 23 Devido a esse fato e à ausência de emissão de radiação, Yildiz et al. 19 recomendam o uso de ultrassonografia de forma alternativa à fluoroscopia.

É importante ressaltar também que os 2 pacientes que não responderam ao tratamento (casos 4 e 12) apresentavam, um grau de artrose máxima na escala de KL (grau 5), bem como a deformidade em varo e flexo. Além disso, 1 dos pacientes apresentava obesidade mórbida (IMC > 40 Kg/m ^2^ ) na ocasião. A soma dessas variáveis pode ter contribuído para um fator de confusão do estudo e resposta zero ao tratamento, conforme evidenciado pelas Tabelas 1 e 2 . Portanto, nos casos de pacientes com deformidades severas e combinadas, bem como IMC muito elevado, esse procedimento deve ser visto com cautela, devido à possibilidade de insucesso.

**Tabela 2:** Pontuações na Escala Numérica Visual (ENV) pré e pós-procedimento

Casos	ENV pré-procedimento	ENV no 1° mês do procedimento	ENV no 3° mês do procedimento	ENV no 6° mês do procedimento
1	8	2	3	4
2	7	2	4	7
3	8	2	3	8
4	8	8	8	8
5	9	3	4	9
6	8	3	3	4
7	8	4	5	8
8	7	2	4	7
9	8	2	2	8
10	9	2	3	9
11	9	3	5	9
12	9	9	9	9
13	8	2	4	8
14	9	2	5	9
15	9	2	7	9
16	9	1	3	4
17	7	2	2	3
18	8	1	3	8
19	8	2	4	4
20	8	2	2	8

Também foi observado que, no sexto mês, 16 (80%) pacientes voltaram a sentir o mesmo nível de dor que sentiam antes do procedimento. Conforme já mencionado, o bloqueio químico é reversível e depende do estímulo doloroso e da cascata de inflamação gerada em nível condral. Nesses casos, é importante enfatizar que todos os pacientes devem realizar fisioterapia antes e depois do procedimento, com o intuito de melhorar a qualidade muscular durante o tratamento e garantir maior durabilidade da analgesia do procedimento.

Embora o presente estudo tenha certas limitações, algumas considerações interessantes devem ser feitas: O BFNG com anestésico local e corticoide foi eficaz para a maioria dos pacientes, que apresentaram melhora do quadro álgico por um período prolongado (superior a 3 meses). Isso ocorre devido ao fato de o BFNG atuar tanto localmente como sistemicamente por influência nas vias moduladoras centrais da dor.

Além disso, o BNFG pode ser importante inicialmente como um bloqueio de teste, no qual se espera melhora imediata do componente álgico (devido à ação imediata do anestésico local) e também no longo prazo (3 meses). Nos casos de resposta favorável, após esse período esperado, o profissional pode considerar o teste positivo e indicar um bloqueio mais prolongado, como a neurólise por fenol ou a ablação por radiofrequência, por exemplo.

## Conclusão

O BFNG é o método eficaz no tratamento da dor crônica relacionada a OAJ, e resulta em melhora da dor com base na ENV. Pode ser utilizado com intuito adjuvante de melhora da qualidade de vida dos pacientes e como parte do arsenal terapêutico para possibilitar a realização de fisioterapia e atividade física com mais eficácia, o que contribui para a melhora dos sintomas da OAJ. No entanto, o BNFG deve ser visto com cautela em pacientes com deformidades avançadas e combinadas, como as em varo e flexo, bem como naqueles com IMC acima de 40 Kg/m ^2^ .
